# Assessing the utility of night‐time presentations as a proxy for alcohol‐related harm among young emergency department trauma patients

**DOI:** 10.1111/1742-6723.14294

**Published:** 2023-08-14

**Authors:** Scott A Sims, Gavin Pereira, Daniel M Fatovich, David Preen, Melissa O'Donnell

**Affiliations:** ^1^ School of Population and Global Health The University of Western Australia Perth Western Australia Australia; ^2^ Curtin School of Population Health Curtin University Perth Western Australia Australia; ^3^ Centre for Fertility and Health Norwegian Institute of Public Health Oslo Norway; ^4^ Emergency Medicine, Royal Perth Hospital The University of Western Australia Perth Western Australia Australia; ^5^ Centre for Clinical Research in Emergency Medicine Harry Perkins Institute of Medical Research Perth Western Australia Australia; ^6^ Australian Centre for Child Protection University of South Australia Adelaide South Australia Australia

**Keywords:** alcohol‐related harm, data linkage, night‐time presentations, proxy measure, young trauma patients

## Abstract

**Objective:**

To assess the usefulness of night‐time presentations to measure alcohol‐related harm (ARH) in young trauma patients, aged 12–24 years, attending Western Australian EDs.

**Methods:**

A retrospective longitudinal study examined alcohol‐related ED presentations in Western Australia (WA; 2002–2016) among 12‐ to 24‐year‐olds. Data from the Emergency Department Data Collection, WA State Trauma Registry Database and Hospital Morbidity Data Collection were used to identify ARH through specific codes and text searches. These were compared to ARH estimates based on presentation time. Statistical analysis involved sensitivity and specificity calculations and Cox proportional hazards modelling.

**Results:**

We identified 2644 (17.8%) night‐time presentations as a proxy measure of ARH among the 14 887 presentations of patients aged 12–24 years. This closely matched the 3064 (20.6%) identified as ARH through coding methods. The highest risk for an ARH presentation occurred during the night hours between 00.00 and 04.59 hours. During these hours, the risk was 4.4–5.1 times higher compared to presentations at midday (between 12.00 and 12.59 hours). However, when looking at individual patients, we observed that night‐time presentations were not a strong predictor of ARH (sensitivity: 0.39; positive predictive value: 0.46).

**Conclusions:**

Implementing targeted interventions during night hours could be beneficial in addressing ARH presentations. However, relying solely on the time of presentation as a proxy for ARH is unlikely to effectively identify ARH in young individuals. Instead, the present study emphasises the importance of implementing mandatory data collection strategies in EDs to ensure accurate measurement of ARH cases.

## Graphical abstract

A retrospective study assessed the usefulness of night‐time presentations as a proxy for measuring alcohol‐related harm (ARH) in young trauma patients in Western Australian EDs. Our analysis revealed that whereas a substantial proportion of ARH cases occurred during night hours, relying solely on presentation time was insufficient to accurately identify ARH in individual patients. Our findings emphasise the importance of implementing mandatory data collection strategies in EDs to ensure accurate measurement and targeted interventions for ARH cases.

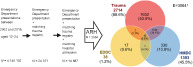

**Key findings**

- Night‐time presentations (between 00.00 and 04.59 hours) for young trauma patients are closely associated with ARH, with the highest risk occuring during these hours.
- However, while night‐time presentations were indicative of ARH risk on a population level, they were not a strong predictor of ARH when considering individual patients.
- Improved data collection methods are required to enhance the understanding and management of ARH in the dynamic ED environment.

## Introduction

Alcohol‐related harm (ARH) is a significant cause of ED visits among young people in Australia,^1^, ^2^, ^3^ with risky drinking behaviour leading to acute alcohol‐related injuries, especially in younger individuals.^4^, ^5^ It is also common for ARH to coexist with mental illness and illicit drug use, further highlighting its significant impact on health.^6^ However, the extent of ARH is often underestimated in routine ED administrative data,^7^ with estimates ranging between 0.8 and 9% of total presentations.^8^ In contrast, survey data indicates that ARH can account for up to 38% of presentations to some Australasian EDs.^1^

Accurate measurement of ARH is crucial for developing effective public health strategies. Unfortunately, EDs, despite being at the forefront of ARH management, face challenges in capturing comprehensive ARH data.^7^, ^9^, ^10^ Current collection methods are inadequate, and Australian EDs are not obligated to screen or collect alcohol‐related presentation data. As a result, alternative measures, such as using time of presentation, have been developed to estimate ARH. These measures suggest that ARH presentations are more likely to occur during night‐time hours, particularly on weekends.^11^, ^12^

Although hospital admissions and trauma registries provide more comprehensive information on ARH, ED data alone lacks this level of detail.^10^, ^13^ To overcome this limitation, the present study aimed to utilise linked data from ED presentations, trauma registries, and inpatient records to identify ARH presentations among young people in Western Australia (WA). By comparing the time‐based measures of ARH in ED with the more complete information obtained through linked data, the study aimed to assess the usefulness of night‐time presentations as a proxy measure for ARH. The study focuses on individuals aged 12–24 years, a critical period characterised by heavy episodic drinking and where interventions for harm minimisation and prevention may be effective.

## Methods

### Design and cohort selection

**Figure 1:**
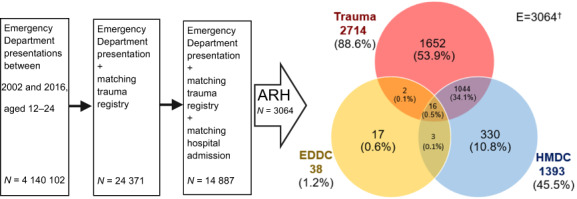
Study cohort selection for people aged 12–24 who presented to an ED between 2002 and 2016. †Venn diagram of alcohol‐related harm (ARH) by data source for patients having Emergency Department Data Collection (EDDC), Trauma Registry, and Hospital Morbidity Data Collection (HMDC) records for the same episode of care.

The present study employed a retrospective longitudinal design to examine individuals aged 12–24 years who had alcohol‐related ED presentations in WA between 2002 and 2016. To gather the necessary data to identify ARH, we utilised three sources: the Emergency Department Data Collection (EDDC), the WA State Trauma Registry Database (Trauma Registry) and the Hospital Morbidity Data Collection (HMDC). Data linkage was performed by the Data Linkage Branch of the WA Department of Health, employing probabilistic matching with clerical review.^14^ For cohort selection, we identified patients whose EDDC presentation date and Trauma Registry arrival date aligned on the same day. Furthermore, these patients had subsequent hospital admissions within 1 day of their ED presentation (Fig. 1).

### Identification of ARH using coded data

To identify ARH, we searched for specific codes related to alcohol in various data fields (Tables [Link], [Link]). A presentation was considered alcohol‐related if an alcohol‐related ICD‐10 code was recorded as the patient's principal diagnosis. We also checked the ‘Presenting Problem’ field, which indicates the primary symptom upon presentation, for alcohol‐related symptom codes. Furthermore, we searched the ‘Diagnosis at Discharge’ and ‘Presenting Complaint’ text fields for the terms ‘alcohol’ or ‘intoxication’. For hospital admissions, we examined the principal diagnosis field, 20 additional diagnosis fields and external cause fields for alcohol‐related ICD‐10 codes. In the Trauma Registry data, we counted cases of ARH if the data item ‘Had Alcohol in Last 12 Hours’ was flagged.

### Estimation of ARH using presentation time

We assessed two presentation time‐based estimates of ARH. The ‘night’ measure included all ED presentations occurring between 00.00 and 04.59 hours from Monday to Sunday. The ‘Weekend night’ measure comprised presentations during the same time frame on Saturday, Sunday and Monday. This method of estimating ARH based on presentation time was initially developed and validated by Young *et al*. in international and Australian EDs.^11^ Although it may not be the optimal method for identifying alcohol‐related injuries in all populations (e.g. not validated for Aboriginal Australians and limited reliability in identifying injury‐related presentations based on primary diagnosis), it is commonly regarded as the most reliable approach.^12^, ^15^

### Cohort characteristics

We collected individual characteristics such as age (12–17 or 18–24 years), sex (male or female) and Aboriginal and Torres Strait Islander status (referred to as Aboriginality). Aboriginality was determined using a validated algorithm by the WA Data Linkage Branch, which analysed records from various WA government administrative datasets where Aboriginal status was recorded.^16^ We identified mental illness and illicit drug use based on specific ICD‐10‐AM diagnosis code categories established in previous research.^7^, ^16^, ^17^ Hospitalisations related to mental illness included any admission or presentation assigned an ICD code indicating a mental and behavioural disorder, excluding cases of deliberate self‐harm involving alcohol, substances and self‐poisoning. Deliberate self‐harm by poisoning was categorised as ARH to avoid duplication in the analysis. Hospitalisations associated with illicit drugs encompassed any ICD code pertaining to cannabis, opioids, methamphetamine, hallucinogens (including lysergide) and cocaine.

### Statistical analysis

Sensitivity and specificity were calculated for alcohol involvement and time of presentation to assess predictive ability. We used multivariate Cox proportional hazards modelling to examine the association between night‐time presentations and coded presentations related to ARH in patients who had an ED presentation, trauma registration, and hospital admission record for the same episode of care. To account for patients with recurrent ARH hospitalisations, we employed a counting process approach. This means that if a patient experienced multiple admissions for ARH, each admission and separation date was included as a separate event in the analysis. The time intervals between these admission and separation dates were considered as separate follow‐up periods for each patient. The patients' age in months was used as the basis for measuring time. This allowed us to track the progression of time by looking at the patients' age at each follow up. It also allowed us to account for any age restrictions or limitations in our analysis. We excluded inter‐hospital transfers to avoid counting them as readmissions. An event was defined as an ARH presentation, with remaining observations classified as not having occurred and hence censored. Follow‐up time was from 12 years of age and censored at either 25 years of age, date of death, or the end of the study period, whichever occurred first.

To assess the association between covariates and ARH, we provided coefficient estimates along with robust standard errors as hazard ratios (HRs), accompanied by Wald 95% confidence intervals (CIs). To confirm the assumption of proportional hazards, we calculated Kaplan–Meier survivor function estimates and performed log‐rank tests. Most categorical covariates exhibited proportional hazards, except for socioeconomic status (SES) and remoteness area, which were both recategorised to satisfy the assumption.

In an additional Cox model, we replaced the dichotomous night‐time presentation covariate with an hour‐based covariate. All models were adjusted for sex, Aboriginality, SES, remoteness area, mental illness and illicit drug comorbidities, time of day and day of the week. Analyses were conducted with SAS V9.4 (SAS Institute, Cary, NC, USA).

### Ethics

Ethics approval for the present study was obtained from the WA Aboriginal Health Ethics Committee (Ref: 755), the University of WA Human Research Ethics Committee (Ref: RA/4/1/8822) and the Department of Health WA Human Research Ethics Committee (Ref: 2016/55).

## Results

### Identification of ARH in ED trauma patients

We identified 3064 ARH cases (20.6%) among the 14 887 presentations of young people aged 12–24 years between 2002 and 2016 in WA with records in all three data sets (EDDC, HMDC and Trauma Registry) for the same episode of care (Fig. 1). Of the ARH cases, 2714 (88.6%) were identified in the Trauma Registry, 1393 (45.5%) in HMDC, whereas only 38 (1.2%) were identified from the EDDC collection. Only 16 (0.5%) of these presentations had ARH identified in all three data sources (Fig. 1). In terms of populations by data source, 1% of all ED presentations (*n* = 23 969), 17.8% of Trauma Registry records (*n* = 4667) and 3.5% of all hospital admissions (*n* = 40 212) were identified as alcohol‐related (Table S3).

Table 1 presents the proportions of alcohol‐related cases identified using coding, night‐time presentations and weekend night presentations for young people aged 12–24 years who presented to EDs between 2002 and 2016 and had a subsequent trauma registration and hospital admission. Among the total presentations (*n* = 14 887), night‐time presentations comprised 17.8% (*n* = 2644). This proportion was found to be the closest proxy for ARH when compared to the coding method, with 20.6% (*n* = 3064) of cases diagnosed as alcohol‐related. In comparison to coding for ARH, the weekend night measure further underestimated ARH (7.5%). The main difference between the night‐time and code methods was for Aboriginality, where 33.9% of Aboriginal people had an alcohol‐related code. This dropped to 19.8% when using presentations at night to estimate ARH. Female patients had fewer presentations identified as ARH than males across all measures, with 17.5% of female presentations having ARH codes compared to 21.4% for males. The proportion of alcohol‐related cases for all methods tends to decrease with increasing socioeconomic advantage, with the least advantaged group having the highest proportion. Major trauma cases with an injury severity score (ISS) >15 have a higher proportion of alcohol‐related cases (32.9%) compared to non‐major trauma cases (19.1%). This difference was not as pronounced for weekend night presentations (10.3% *vs* 7.1%, respectively).

**TABLE 1:** Proportion of alcohol‐related cases identified by coding, night presentations and weekend night presentations for young people aged 12–24 years presenting to EDs between 2002 and 2016‡

	ARH code		Night presentations†		Weekend night presentations†		Total
Characteristic	*n*	%	*n*	%	*n*	%	*N*
Age group							
12–17 years	483	8.3	739	12.7	337	5.8	5831
18–24 years	2581	28.5	1905	21.0	777	8.6	9056
Sex							
Female	552	17.5	529	16.7	220	7.0	3162
Male	2512	21.4	2115	18.0	894	7.6	11 725
Aboriginality							
Non‐Aboriginal	2497	18.9	2313	17.5	967	7.3	13 214
Aboriginal	567	33.9	331	19.8	147	8.8	1673
Trauma type							
Major (ISS >15)	535	32.9	425	26.2	168	10.3	1624
Non‐major (ISS <16)	2529	19.1	2219	16.7	946	7.1	13 263
Socioeconomic status							
Missing	235	25.0	211	22.4	88	9.3	942
1 Least advantaged	922	23.9	752	19.5	313	8.1	3861
2	634	20.6	551	17.9	228	7.4	3078
3	474	18.2	454	17.4	192	7.4	2605
4	440	18.1	389	16.0	172	7.1	2435
5 Most advantaged	359	18.3	287	14.6	121	6.2	1966
Remoteness area							
Missing	235	25.0	211	22.4	88	9.3	942
Major cities	2098	19.8	1761	16.6	765	7.2	10 615
Regional	415	20.5	435	21.5	161	8.0	2026
Remote	316	24.2	237	18.2	100	7.7	1304
Total	3064	20.6	2644	17.8	1114	7.5	14 887

† Night: 00.00 to 04.59 hours; weekend night: 00.00 to 04.59 hours on Saturday, Sunday and Monday.

‡ Cohort includes only presentations with a corresponding Trauma Registry and hospital admission for the same episode of care.

ARH, alcohol‐related harm; ISS, injury severity score.

### Accuracy of time‐based measures of ARH

The calculation of sensitivity and specificity was performed on the cohort between time‐based measures and coding methods (Table 2). Overall sensitivity for identifying ARH was low, with the highest sensitivity for predicting alcohol involvement via coding methods achieved using any night presentations for ARH (39%). Positive predictive values (PPVs) were also the highest for any night presentations (46%). Specificity was lowest for any night presentation times (88%) and negative predictive values were above 81%. There was, therefore, minimal concordance for ARH identification across the three data sources.

**TABLE 2:** Contingency table comparing alcohol‐related harm (ARH) coding to any night‐time presentations and weekend night presentations for young people aged 12–24 years presenting to EDs between 2002 and 2016†,‡

	ARH code		Total				
	No	Yes					
Any night presentation§							
No	10 384	1859	12 243	Concordance rate	0.78		
Yes	1439	1205	2644	Sensitivity	0.39	Positive predictive value	0.46
Total	11 823	3064	14 887	Specificity	0.88	Negative predictive value	0.85
Weekend night presentation§							
No	11 203	2570	13 773	Concordance rate	0.79		
Yes	620	494	1114	Sensitivity	0.16	Positive predictive value	0.44
Total	11 823	3064	14 887	Specificity	0.95	Negative predictive value	0.81

† Sensitivity (positive per cent agreement): proportion of ARH presentations for which the time‐based measure was coded as ARH; positive predictive value: proportion of time‐based measure for which the presentation was coded as ARH; specificity (negative per cent agreement): proportion of presentations that were not coded as ARH for which the time‐based measure was also not ARH; negative predictive value: the proportion of time‐based presentations that were not ARH for which the presentation was also not ARH. The reference standard for ARH was based on the coding method.

‡ Cohort includes only presentations with a corresponding Trauma Registry and hospital admission for the same episode of care.

§ Night: 00.00 to 04.59 hours; weekend night: 00.00 to 04.59 hours on Saturday, Sunday and Monday.

### Risk factors of ED trauma patients with ARH

Table 3 displays the factors associated with ARH presentations adjusted for all variables in the Cox regression model. The risk of having alcohol involvement recorded (either via code or clinical text) was 17% higher in males than females presenting with ARH (HR 1.17; 95% CI 1.07–1.27), whereas Aboriginal people were at 80% higher risk of this outcome than non‐Aboriginal people (HR 1.80; 95% CI 1.65–1.96). Major trauma (ISS >15) admission patients were 43% more at risk of presenting with ARH than young people with non‐major trauma (HR 1.43; 95% CI 1.31–1.55). People presenting with ARH were also 2.5 times more likely to have illicit drug involvement than patients without mention of ARH in their medical records (HR 2.53; 95% CI 2.32–2.76). Night‐time presentations were 2.5 times as likely to have a presentation coded as alcohol‐related compared to day‐time presentations (HR 2.54; 95% CI 2.38–2.71). However, where people resided and what day they presented to ED had little association with the risk of presenting with ARH. In addition, ARH presentations had a non‐significant association with being mental illness‐related (HR 1.05; 95% CI 0.87–1.27).

**TABLE 3:** Risk of alcohol‐related presentations for 12‐ to 24‐year‐old people having an ED, trauma and hospital admission for the same episode of care, 2002–2016

Characteristic	Unadjusted HR	Adjusted HR†
	(95% CI)	(95% CI)
Sex		
Male	1.15 (1.06–1.25)**	1.17 (1.07–1.27)***
Female	REF	REF
Aboriginality		
Aboriginal	1.90 (1.76–2.05)***	1.80 (1.65–1.96)***
Non‐Aboriginal	REF	REF
Trauma type		
Major (ISS >15)	1.62 (1.49–1.76)***	1.43 (1.31–1.55)***
Non‐major (ISS <16)	REF	REF
Socioeconomic status		
Lower 20% disadvantaged	1.25 (1.17–1.34)***	1.10 (1.03–1.19)***
Upper 80%	REF	REF
Remoteness area		
Major cities	REF	REF
Regional	1.11 (1.02–1.22)*	0.99 (0.90–1.09)
Remote	1.23 (1.11–1.37)***	0.96 (0.86–1.07)
Illicit drug‐related‡		
Yes	3.21 (2.98–3.46)***	2.53 (2.32–2.76)***
No	REF	REF
Mental illness‐related‡		
Yes	1.12 (0.92–1.36)	1.05 (0.87–1.27)
No	REF	REF
Day of presentation		
Sunday	1.08 (0.95–1.21)	1.09 (0.97–1.23)
Monday	REF	REF
Tuesday	1.05 (0.93–1.18)	1.02 (0.90–1.15)
Wednesday	1.06 (0.94–1.20)	1.08 (0.96–1.22)
Thursday	1.15 (1.02–1.29)*	1.10 (0.98–1.24)
Friday	1.03 (0.91–1.16)	1.05 (0.93–1.18)
Saturday	1.06 (0.94–1.19)	1.06 (0.94–1.19)
Time of day		
00.00 to 05.00 hours	2.74 (2.58–2.92)***	2.54 (2.38–2.71)***
05.00 to 00.00 hours	REF	REF

* *P* < 0.05.

** *P* < 0.01.

*** *P* < 0.001.

† Multivariate Cox regression model is adjusted for characteristics.

‡ Related to presenting episode of care only.

CI, confidence interval; HR, hazard ratio; ISS, injury severity score; REF, reference category.

**Figure 2:**
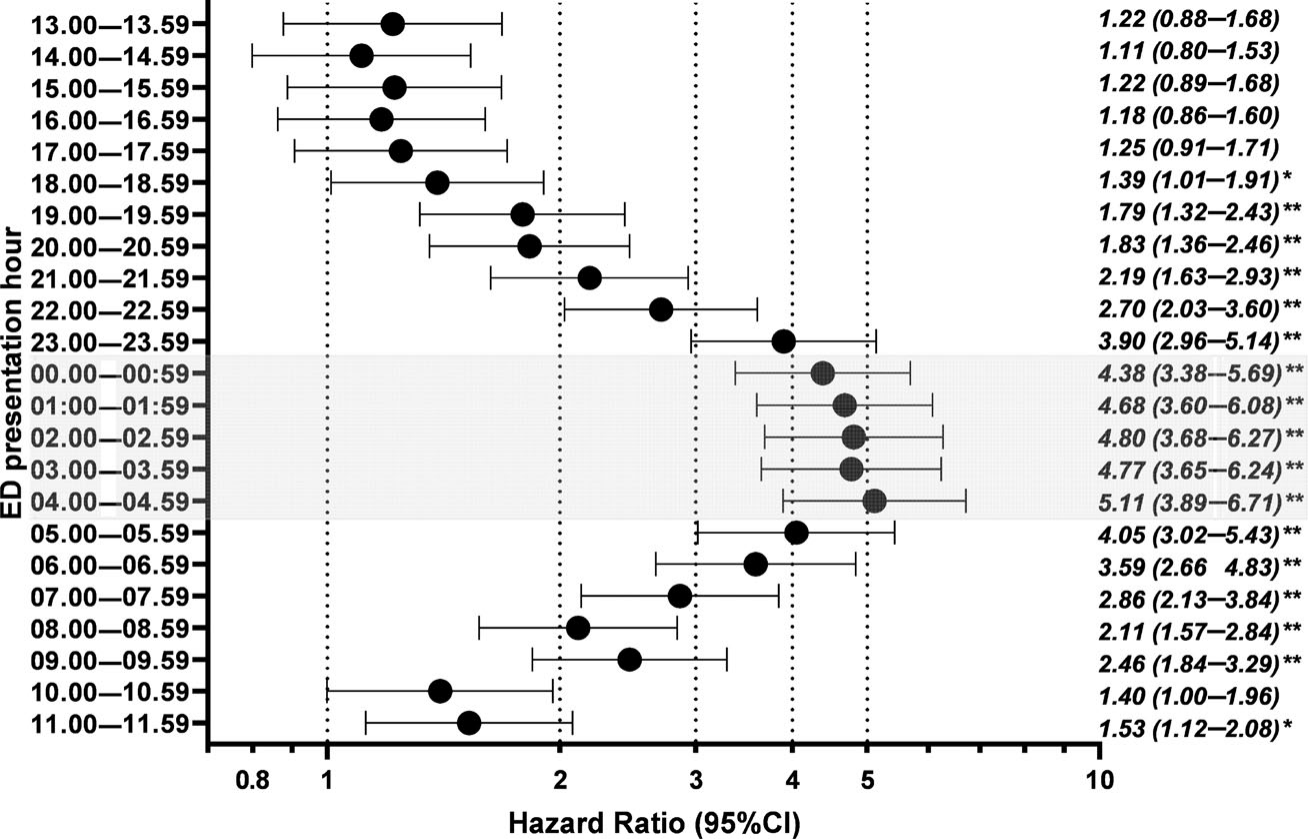
Hourly risk of alcohol‐related ED presentations for patients aged 12–24 with both a trauma and hospital admission, 2002–2016†. †Based on adjusted hazard ratio (95%) estimates, reference category defined as 12.00 to 12.59 hours; *P < 0.05; **P < 0.0001; shaded hazard ratios = night‐time presentations; *X*‐axis presented on a logarithmic scale.

Figure 2 shows results from the multivariate Cox model examining the variation between each hour of presentation and its association with ARH presentations. The five largest effect sizes corresponded to the night‐time period, with the highest risk of ARH associated with presenting between 04.00 and 04.59 hours, 5.1 times higher than those presenting between 12.00 and 12.59 hours (HR 5.11; 95% CI 3.89–6.71). No significant association was found for presentations between 13.00 and 17.59 hours and the presentation being alcohol‐related.

## Discussion

This research aimed to compare the identification of ARH in young patients admitted to hospital EDs with injury. We used standard clinical coding and considered the time of presentation to estimate ARH. Our findings indicate that the highest risk of ARH presentations occurs during night hours. However, when looking at individual patients, using presentation time alone is not a reliable predictor of ARH. This is because there are multiple factors contributing to ARH presentations that are not related to the specific time of presentation and these are not routinely collected.

Identifying the night hours between 00.00 and 04.59 hours as the highest risk period for ARH presentations could help inform targeted interventions during these times, which aligns with findings from other studies.^11^, ^13^, ^17^, ^18^ However, it is important to approach the conclusion drawn from a recent report commissioned and published by the Brewers' Association with caution, considering the potential conflict of interest associated with its provenance.^19^ This report suggests a small and declining share of ED presentations in Australia can be attributed to ARH. It is worth noting that this conclusion is based on inadequate diagnostic data from EDs, which is also used by the Australian government for official statistics. In contrast, our findings, along with previous research, indicate that relying solely on Australian ED data underestimates the prevalence of ARH presentations.^7^, ^13^, ^20^

Despite the common association between ARH and mental illness, our findings show that mental illness‐related presentations were not significantly associated with ARH in trauma patients who were hospitalised.^21^ These patients may have unique characteristics compared to the general population presenting to EDs. For example, males historically access mental health services less frequently than females.^22^, ^23^ Considering that most of this population were male (79%) and more likely to be Aboriginal and reside in disadvantaged areas, it is possible that they seek mental health services less often. Further research is needed to understand these differences compared to non‐trauma patients who are not hospitalised.

Efforts are being made to address the deficiencies in current ED collections for monitoring the true burden of ARH. In Australia, monitoring ARH is a key priority in the National Alcohol Strategy 2019–2028, which aims to improve community safety and amenity. This includes estimating alcohol‐related ED presentations on Friday, Saturday and Sunday nights per 1000 persons.^24^ In comparison, New Zealand has mandated the recording of ‘alcohol involved’ presentations in their National Non‐admitted Patient Collection, following recommendations from the ACEM.^25^ We recommend that Australia adopt a similar approach in the National Minimum Dataset for National Non‐admitted Patient Emergency Department Care.^26^, ^27^

Although the coding and text methods we utilised can be relatively automated, they are limited by poor data quality and lengthy approval processes. Low sensitivity and PPVs suggest that EDs need better support and funding to improve their diagnostic coding practices for accurately capturing and reporting ARH cases. This may involve providing additional training to staff for accurately identifying and coding cases of ARH. However, collecting high‐quality ARH data is likely to require innovative methods that can be implemented in busy EDs with high staff turnover.^1^, ^28^ One such approach is the use of machine learning and natural language processing on underutilised ED data, including free text fields recorded by clinicians such as triage notes, ED medical assessments and treatment information. By improving data quality and implementing these advancements, we can enhance our understanding of ARH and support effective interventions to mitigate its impact on public health and safety.

### Limitations

The present study had several limitations that should be acknowledged. First, linking multiple sources to the same episode of care may introduce bias towards a particular type of person, which may not be generalisable to the entire population of ED presentations. Instead, our findings are more applicable to ‘high‐risk’ ED trauma patients who were serious enough to be hospitalised. Second, we assumed hospital admissions occurring within 1 day of a presentation belonged to the same episode of care based on national hospital statistics.^29^ Finally, patients admitted for injuries where only the perpetrator had been affected by alcohol are not coded as ARH.

## Conclusions

Our findings indicate the use of time‐based proxies alone is unlikely to be sufficient for effectively identifying and managing alcohol‐related presentations in EDs. A multifaceted approach is needed which may require changes in policies and practices related to screening, diagnostic coding, and targeted interventions for patients who have experienced ARH. It is crucial to address the limitations in data collection methods and support EDs with better resources and funding. By adopting innovative techniques such as machine learning and natural language processing, we can enhance the quality and comprehensiveness of ARH data in the busy and dynamic ED environment.

## Supporting information


**Table S1.** Alcohol‐related harm ICD‐10‐AM code categories and descriptions.

**Table S2.** Alcohol‐related harm symptom code categories, names, and subgroup level.

**Table S3.** Comparison of alcohol‐related harm identified separately in EDDC, HMDC and Trauma Registry data collections between 2002–2016 for young people aged 12‐24.

## Data Availability

Data available on request due to privacy/ethical restrictions.
