# Supplementary material for: Assessing the utility of night‐time presentations as a proxy for alcohol‐related harm among young emergency department trauma patients
Source: Emerg Med Australas. 2023 Aug 14;36(1):47–54. doi: 10.1111/1742-6723.14294 (PMC10952259; doi:10.1111/1742-6723.14294)
Supplement: Supplementary file 1 — Table S1. Alcohol‐related harm ICD‐10‐AM code categories and descriptions. [file EMM-36-47-s003.docx]

Table S1. *Alcohol-related harm ICD-10-AM code categories and descriptions*

| ICD-10-AM | Description of alcohol-related hospitalisations |
| --- | --- |
| E24.4 | Alcohol-induced pseudo-Cushing's syndrome |
| E52 | Alcoholic pellagra *[if assigned with a code F10.x]* |
| F10 | Mental and behavioural disorders due to use of alcohol |
| G31.2 | Degeneration of nervous system due to alcohol |
| G40.5 | Epileptic seizures related to alcohol |
| G62.1 | Alcoholic polyneuropathy |
| G72.1 | Alcoholic myopathy |
| I42.6 | Alcoholic cardiomyopathy |
| K29.2 | Alcoholic gastritis |
| K70 | Alcoholic liver disease |
| K85 | Alcohol pancreatitis *[if assigned with a code F10.x]* *[1999-Jul-01 to 2006-Jun-30]* |
| K85.2 | Alcohol-induced acute pancreatitis *[2006-Jul-01 onwards]* |
| K86.0 | Alcohol-induced chronic pancreatitis |
| O35.4 | Maternal care for (suspected) damage to fetus from alcohol |
| R78.0 | Finding of alcohol in blood |
| T51.0 | Toxic effect: Ethanol |
| T51.1 | Toxic effect: Methanol |
| T51.9 | Toxic effect: Alcohol, unspecified |
| X45 | Accidental poisoning by and exposure to alcohol |
| X65 | Intentional self-poisoning by and exposure to alcohol |
| Y15 | Poisoning by and exposure to alcohol, undetermined intent |
| Y90 | Evidence of alcohol involvement determined by blood alcohol level *[excl. Y90.9]* |
| Z71.4 | Alcohol abuse counselling and surveillance |
| Z72.1 | Problems related to lifestyle - Alcohol use |

Note: Codes selected as being appropriate for harm due to consumption of alcohol were based on clinical coding advice received from the Western Australian Clinical Coding Authority (WACCA). Z codes related to historic conditions were excluded. ICD-10-AM refers to the International Statistical Classification of Diseases and Related Health Problems, Tenth Revision, Australian Modification
