# Supplementary material for: Assessing the utility of night‐time presentations as a proxy for alcohol‐related harm among young emergency department trauma patients
Source: Emerg Med Australas. 2023 Aug 14;36(1):47–54. doi: 10.1111/1742-6723.14294 (PMC10952259; doi:10.1111/1742-6723.14294)
Supplement: Supplementary file 2 — Table S2. Alcohol‐related harm symptom code categories, names, and subgroup level. [file EMM-36-47-s001.docx]

Table S2. *Alcohol-related harm symptom code categories, names, and subgroup level*

| ED symptom code | ED symptom name | Level |
| --- | --- | --- |
| E0000 | DRUG / ALCOHOL USE | 1 |
| EA000 | ALCOHOL INTOXICATION | 2 |
| EB000 | ALCOHOL WITHDRAWAL | 2 |
| ECA00 | DRUG OVERDOSE AND ALCOHOL | 3 |
| EF000 | ALCOHOL | 2 |
| EFA00 | ALCOHOL INTOXICATION | 3 |
| EFB00 | ALCOHOL WITHDRAWAL | 3 |
| EHBH4 | ALCOHOL SELF HARM | 5 |
| EKBA0 | DRUG OVERDOSE & ALCOHOL | 4 |
