# Supplementary material for: Assessing the utility of night‐time presentations as a proxy for alcohol‐related harm among young emergency department trauma patients
Source: Emerg Med Australas. 2023 Aug 14;36(1):47–54. doi: 10.1111/1742-6723.14294 (PMC10952259; doi:10.1111/1742-6723.14294)
Supplement: Supplementary file 3 — Table S3. Comparison of alcohol‐related harm identified separately in EDDC, HMDC and Trauma Registry data collections between 2002–2016 for young people aged 12‐24. [file EMM-36-47-s002.docx]

**Table S3:** *Comparison of alcohol-related harm (ARH) identified separately in EDDC, HMDC and Trauma Registry data collections between 2002—2016 for young people aged 12-24*

|  | Emergency presentations (EDDC) | | | |  | Trauma Registrations | | | |  | Hospital admissions (HMDC) | |
| --- | --- | --- | --- | --- | --- | --- | --- | --- | --- | --- | --- | --- |
| Characteristic | Alcohol coding | | Between 00:00-04:49 (night) | |  | Alcohol coding | | Between 00:00-04:59 (night) | |  | Alcohol coding | |
|  | n | % | n | % |  | n | % | n | % |  | n | % |
| Age group |  |  |  |  |  |  |  |  |  |  |  |  |
| 12-17 years | 6859 | 0.3 | 52242 | 2.3 |  | 773 | 3.0 | 853 | 3.3 |  | 9161 | 0.8 |
| 18-24 years | 17110 | 0.7 | 147128 | 6.4 |  | 3894 | 14.9 | 2437 | 9.3 |  | 31051 | 2.7 |
| Gender |  |  |  |  |  |  |  |  |  |  |  |  |
| Female | 12174 | 0.5 | 98822 | 4.3 |  | 833 | 3.2 | 690 | 2.6 |  | 18829 | 1.6 |
| Male | 11794 | 0.5 | 100531 | 4.4 |  | 3834 | 14.6 | 2600 | 9.9 |  | 21383 | 1.9 |
| Aboriginality |  |  |  |  |  |  |  |  |  |  |  |  |
| Non-Aboriginal | 21186 | 0.9 | 167885 | 7.3 |  | 3707 | 14.1 | 2828 | 10.8 |  | 30322 | 2.6 |
| Aboriginal | 2783 | 0.1 | 31485 | 1.4 |  | 960 | 3.7 | 462 | 1.8 |  | 9890 | 0.9 |
| Socio-economic status |  |  |  |  |  |  |  |  |  |  |  |  |
| Missing | 1738 | 0.1 | 10910 | 0.5 |  | 309 | 1.2 | 250 | 1.0 |  | 1685 | 0.1 |
| 1 Least advantaged | 5276 | 0.2 | 59386 | 2.6 |  | 1372 | 5.2 | 862 | 3.3 |  | 12582 | 1.1 |
| 2 | 4595 | 0.2 | 42195 | 1.8 |  | 987 | 3.8 | 699 | 2.7 |  | 8560 | 0.7 |
| 3 | 4005 | 0.2 | 33817 | 1.5 |  | 762 | 2.9 | 581 | 2.2 |  | 6227 | 0.5 |
| 4 | 4065 | 0.2 | 28771 | 1.3 |  | 680 | 2.6 | 491 | 1.9 |  | 5842 | 0.5 |
| 5 Most advantaged | 4290 | 0.2 | 24291 | 1.1 |  | 557 | 2.1 | 407 | 1.6 |  | 5316 | 0.5 |
| Remoteness Area |  |  |  |  |  |  |  |  |  |  |  |  |
| Missing | 1738 | 0.1 | 10910 | 0.5 |  | 309 | 1.2 | 250 | 1.0 |  | 1685 | 0.1 |
| Major Cities | 19456 | 0.8 | 125829 | 5.5 |  | 3192 | 12.2 | 2280 | 8.7 |  | 25613 | 2.2 |
| Regional | 2438 | 0.1 | 38754 | 1.7 |  | 687 | 2.6 | 483 | 1.8 |  | 6964 | 0.6 |
| Remote | 337 | 0.0 | 23877 | 1.0 |  | 479 | 1.8 | 277 | 1.1 |  | 5950 | 0.5 |
| Day of presentation |  |  |  |  |  |  |  |  |  |  |  |  |
| Sunday | 3485 | 0.2 | 28223 | 1.2 |  | 1040 | 4.0 | 769 | 2.9 |  | 7829 | 0.7 |
| Monday | 3367 | 0.1 | 27200 | 1.2 |  | 543 | 2.1 | 403 | 1.5 |  | 4915 | 0.4 |
| Tuesday | 3429 | 0.1 | 29081 | 1.3 |  | 464 | 1.8 | 365 | 1.4 |  | 4534 | 0.4 |
| Wednesday | 3426 | 0.1 | 29036 | 1.3 |  | 446 | 1.7 | 340 | 1.3 |  | 4626 | 0.4 |
| Thursday | 3377 | 0.1 | 28834 | 1.3 |  | 519 | 2.0 | 362 | 1.4 |  | 5006 | 0.4 |
| Friday | 3462 | 0.2 | 28766 | 1.3 |  | 586 | 2.2 | 375 | 1.4 |  | 5536 | 0.5 |
| Saturday | 3423 | 0.1 | 28230 | 1.2 |  | 1069 | 4.1 | 676 | 2.6 |  | 7766 | 0.7 |
| Total alcohol | 23969 | 1.0 | 199370 | 8.7 |  | 4667 | 17.8 | 3290 | 12.6 |  | 40212 | 3.5 |
| Total cases | 2289555 |  | 2289555 |  |  | 26198 |  | 26198 |  |  | 1145210 |  |

Note: HMDC = Hospital Morbidity Data Collection; EDDC = Emergency Department Data Collection
